# Repetitive Non–Re-Entrant Ventriculoatrial Synchrony Causing Heart Failure Exacerbation

**DOI:** 10.1016/j.jaccas.2026.107258

**Published:** 2026-05-20

**Authors:** Ufuk Vardar, James F. Howick, Carlos Vergara Sanchez, Jeanwoo Yoo, William Mai, Bridget Lee, Iain Thompson, K.L. Venkatachalam

**Affiliations:** Department of Cardiovascular Medicine, Mayo Clinic, Jacksonville, Florida, USA

**Keywords:** AV delay, heart failure, LRL, PMT, RNRVAS

## Abstract

**Background:**

Repetitive non-reentrant ventriculoatrial synchrony (RNRVAS) is an under-recognized pacemaker-mediated rhythm that can cause atrioventricular (AV) dyssynchrony and heart failure symptoms.

**Case Summary:**

A 65-year-old man with advanced nonischemic cardiomyopathy and dual-chamber implantable cardioverter-defibrillator presented with worsening heart failure symptoms. A 12-lead electrocardiogram (ECG) demonstrated ventricular pacing with retrograde atrial conduction followed by atrial pacing with functional atrial noncapture, consistent with RNRVAS. Device interrogation confirmed frequent automatic mode switching episodes. Targeted device reprogramming, including lowering the lower rate limit and shortening the AV delay restored AV synchrony and resulted in clinical improvement.

**Discussion:**

RNRVAS may evade standard device detection algorithms and present with subtle but clinically significant hemodynamic compromise. Careful ECG analysis can facilitate timely diagnosis and guide simple programming adjustments, with profound symptom improvement.

**Take-Home Message:**

Careful analysis of surface ECGs can reveal RNRVAS and guide simple pacemaker reprogramming that restores AV synchrony and improves heart failure symptoms.

## Visual Summary

**Figure:**
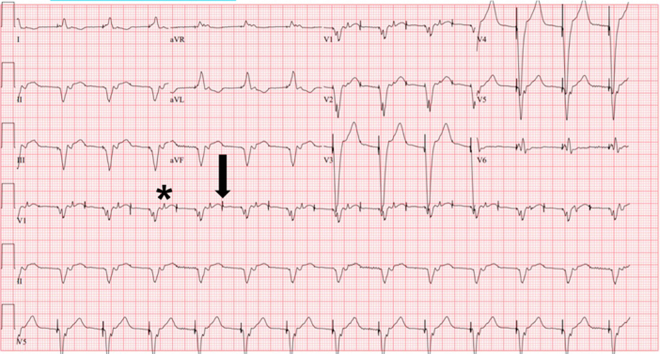

## History of Present Illness

A 65-year-old man with a history of nonischemic cardiomyopathy, with left ventricular ejection fraction of 15% to 20%, atrial fibrillation/flutter, and dual-chamber implantable cardioverter-defibrillator (ICD) (St Jude Medical Ellipse DR, model 2411-36Q) for primary prevention, presented with worsening shortness of breath, bendopnea, abdominal bloating, and lower extremity swelling in the past 2 months, for evaluation for advanced heart failure therapies. He felt fatigued and had 3-pillow orthopnea. Vital signs on arrival included a blood pressure of 85/42 mm Hg, pulse rate of 80 beats/min, respiratory rate of 14 breaths/min, and saturation 95% on room air. Physical examination revealed cachexia, temporal wasting, jugular venous pressure 11 cm from the angle of Louis while sitting upright, palpable liver 1 cm below the right costal margin, and symmetric pitting edema of the legs up to the knees.

**Take-Home Message**

• Recognition of repetitive non-reentrant ventriculoatrial synchrony through careful implantable cardioverter-defibrillator and ECG analysis and targeted device reprogramming can restore atrioventricular synchrony and lead to meaningful symptomatic improvement.

Three months prior, he had a 35-day hospitalization with cardiogenic shock and pulmonary edema that had requiring mechanical ventilation, temporary mechanical circulatory support with a percutaneous microaxial ventricular assist device, and temporary dialysis.

## Previous Medical History

The patient was diagnosed with heart failure 1 year prior to arrival, with a positron emission tomography perfusion scan negative for ischemia/infarction; he underwent atrial flutter and fibrillation ablation complicated by intraoperative perforation requiring surgical window. He also had long-standing hypertension, type 2 diabetes mellitus, hyperlipidemia, chronic kidney disease stage III with prior need for temporary dialysis, moderate mitral and tricuspid regurgitation, and moderate pulmonary hypertension likely secondary to left heart disease.

## Differential Diagnosis

The differential diagnosis for his acute presentation included arrhythmias, coronary artery disease, valvular dysfunction, dietary/medication nonadherence, and pacemaker-mediated cardiomyopathy due to frequent right ventricular pacing and pulmonary hypertension.

## Investigations

A 12-lead electrocardiogram (ECG) showed ventricular pacing with retrograde atrial conduction and atrial pacing with functional atrial noncapture, suggestive of repetitive non-reentrant ventriculoatrial synchrony (RNRVAS).

ICD interrogation was performed, with 152 automatic mode switching (AMS) episodes noted within 3 weeks. The device capture and sensing were working appropriately. Intracardiac device electrograms demonstrated ventricular pacing, with retrograde atrial activity falling within the postventricular atrial refractory period (PVARP) and atrial pacing with functional noncapture (owing to atrial tissue refractoriness from the retrograde P waves), leading to AMS by the device because of perceived atrial tachycardia from the A Pace and A Sense episodes (Figure 1). Retrograde conduction time/ventriculoatrial (VA) interval was measured as 158 ms, also demonstrated in Figure 1. The device was in DDDR mode, with parameters including lower rate limit (LRL) 60 beats/min, maximum tracking rate 100 beats/min, maximum sensor rate 115 beats/min, paced and sensed atrioventricular (AV) delay 300 ms, postventricular atrial blanking 80 ms, and PVARP 300 ms.

**Figure 1:**
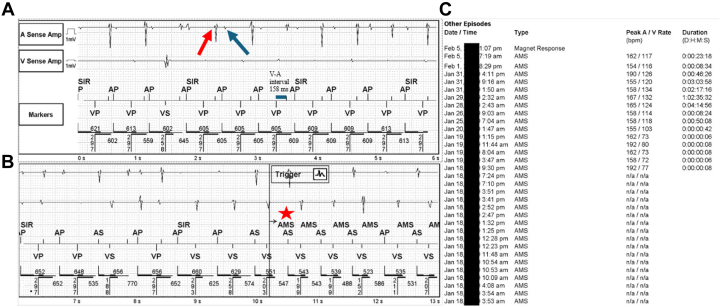
Intracardiac Electrograms From Device Interrogation With Atrial (Top) and Ventricular (Middle) Signals and Marker Channel (Bottom) (A) Ventricular pacing (VP), retrograde atrial activity (red arrow), and atrial pacing (AP) stimulus output with functional noncapture (blue arrow). The ventriculoatrial (VA) interval was 158 ms. (B) Atrial-paced (AP) and ventricular-sensed (VS) rhythm, sensor-indicated rate (SIR) recognition, and automatic mode switching (AMS; red star), alternating from DDDR to DDIR mode. (C) AMS episodes clustered, with the longest duration being 1 day and 2 hours.

Basic metabolic panel showed a blood urea nitrogen of 50 mg/dL, serum creatinine of 2.0 mg/dL, hepatic function panel with total bilirubin 2.1 mg/dL, alanine aminotransferase 118 U/L, aspartate aminotransferase 42 U/L, alkaline phosphatase 139 U/L, albumin 4.0 g/dL, and uric acid 13.5 mg/dL. N-terminal pro–B-type natriuretic peptide was 14,737 pg/mL. Complete blood cell count, thyroid-stimulating hormone, and urine drug screen were unremarkable. Transthoracic echocardiography indicated a left ventricular ejection fraction of 14%, severe generalized hypokinesis, normal right ventricular size with moderate decrease in systolic function, and mild to moderate mitral regurgitation. Coronary angiography revealed mild nonobstructive coronary artery disease. Right heart catheterization showed a systemic blood pressure of 88/74 mm Hg (mean arterial pressure: 79 mm Hg), right atrial pressure of 8 mm Hg, pulmonary artery pressure of 36/20 mm Hg (mean: 25 mm Hg), pulmonary arterial wedge pressure of 20 mm Hg, and cardiac index by assumed Fick method of 1.3 L/min/m^2^.

## Management

The patient's device was reprogrammed to decrease the likelihood of RNRVAS by increasing the VA interval and allowing atrial capture after the atrium had recovered. The LRL was decreased to 50 beats/min, and the AV delay was shortened to 200 ms; these changes were intended to allow atrial recovery after retrograde activation by increasing the VA interval of the device to restore effective atrial capture. Shortening the PVARP (300 ms at baseline) was considered, however shortening it to less than the VA interval of 158 ms would significantly increase the risk of pacemaker-mediated tachycardia (PMT), and so this was deferred. Repeat 12-lead ECG 2 weeks later revealed sinus tachycardia at a heart rate of 127 beats/min with premature ventricular contractions and fusion complexes, left atrial abnormality (>1 mm^2^ terminal negative portion of P wave in V1), and left ventricular hypertrophy per Cornell criteria (R in aVL plus S in V_3_ >28 mm) (Figure 2).

**Figure 2:**
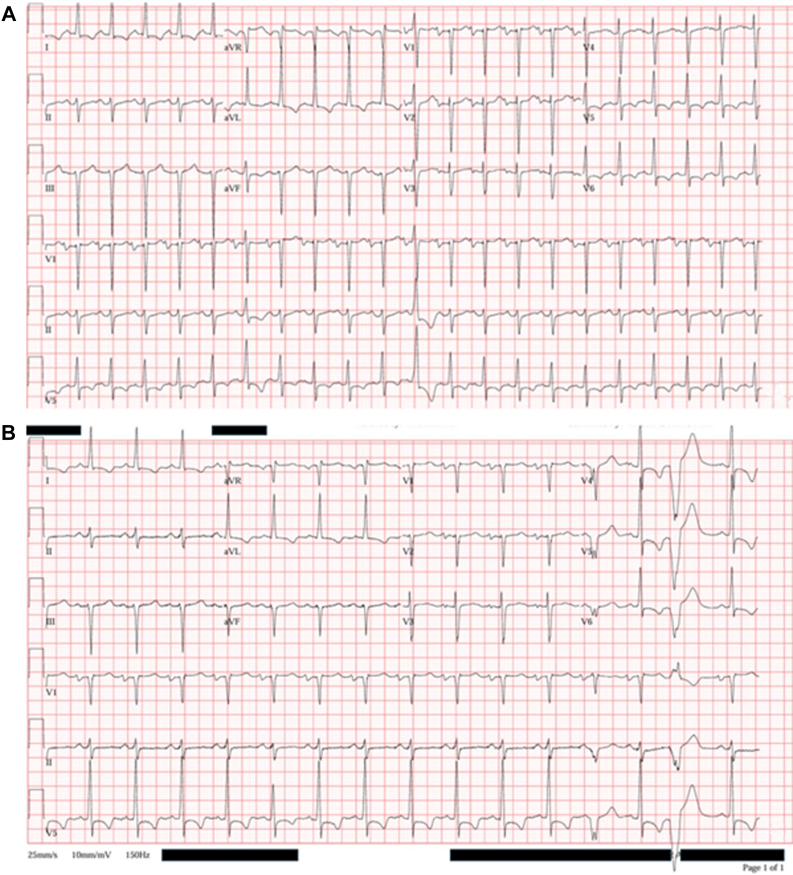
Repeat 12-Lead ECGs (A) Sinus tachycardia (heart rate: 127 beats/min) with PVCs and fusion complexes. (B) Sinus rhythm (heart rate: 93 beats/min) with a single PVC. ECG = electrocardiogram; PVC = premature ventricular contraction.

The patient was started on inotropic therapy with milrinone, received intravenous diuresis for 3 days, and was discharged on home inotropes. Lead upgrade for cardiac resynchronization therapy was not considered given improvement in symptoms and pending heart transplantation.

## Outcome and Follow-Up

After the device reprogramming and inotropic therapy, the patient improved clinically and underwent orthotopic heart transplantation and ICD explantation 4 months later, with no major complications.

## Discussion

RNRVAS is an under-recognized entity that can lead to pacemaker syndrome–like symptoms due to loss of optimal AV synchrony, inappropriate increase in ventricular pacing, and increased incidence of atrial fibrillation.^1^ This macro-reentrant arrhythmia uses ventricular pacing as the anterograde limb along with intact long intrinsic VA conduction as the retrograde limb, similar to PMT.^2^

Retrograde atrial depolarization (ie, premature ventricular contraction as an initiator) falls within the PVARP in RNRVAS (in contrast to “after” PVARP in PMT), leading to “functional” undersensing (failure to sense an event because it is in the refractory period). Atrial pacing is attempted by the pacemaker when the VA interval timer times out, resulting in functional noncapture (failure to capture owing to the refractoriness of the atrial myocardium); the next ventricular pacing interval completes the circuit after paced AV delay.^2^^,^^3^ PMT typically presents with palpitations at higher heart rates (or near the upper tracking rate), and devices have algorithms to recognize it, while RNRVAS may be more subtle and occur at lower rates, potentially making it under-recognized despite causing hemodynamic compromise from AV desynchronization.^4^

Our patient presented with heart failure exacerbation (worsening congestion and perfusion), with frequent episodes of AMS (atrial tachycardia/atrial fibrillation burden: <1%, AMS burden: 7.1%), some represented RNRVAS, while some were true atrial tachycardia/atrial fibrillation episodes, which may be triggered by pacing the atrial in shorter cycle lengths (ie, 300 ms) by the mechanism explained above. In St Jude Medical dual-chamber devices, both atrial pacing and atrial sensing events contribute to the atrial rate and may trigger inappropriate mode switching.^1^ These episodes are stored as atrial high-rate episodes and can be retrieved and reviewed to confirm atrial tachyarrhythmias, as in our case.

A recently recognized similar entity, named “pseudo” RNRVAS, may mimic RNRVAS with the main difference being the absence of VA conduction, with “sinusal” P waves being repetitively trapped in the PVARP.^5^^,^^6^ A 12-lead ECG may be a helpful tool as an adjunctive method of differentiating pseudo-RNRVAS from true RNRVAS, as retrograde P waves from true RNRVAS (atrial exit near the AV node) would have a different morphology from sinus origin (ie, negative in inferior leads); this was demonstrated in our case.

RNRVAS is promoted by high LRL or rapid pacing, long paced and sensed AV intervals, or long PVARP.^1^ Management includes shortening the AV delay and lowering the LRL to lengthen the VA interval and allow the atrial tissue to recover after retrograde atrial activation and be available for atrial capture. Shortening the PVARP may also help by uncovering the retrograde atrial activity, but this leaves the patient vulnerable to PMT. Our patient experienced symptomatic improvement after device reprogramming, which served as an effective bridge therapy while awaiting cardiac transplantation.

As a conceptual framework, RNRVAS might be identifiable by repetitive atrial-sensed–atrial-paced (“AS-AP”) pairs occurring in close temporal proximity in a bigeminal pattern, reflecting functional atrial undersensing followed by functional noncapture. This pattern may help distinguish RNRVAS from true atrial high-rate episodes, which are typically characterized by atrial-sensed events without closely coupled atrial pacing given adherence to the LRL. Recognition of this electrogram signature could, in principle, permit automated responses such as transient lowering of the LRL or shortening of the AV delay to restore atrial capture and AV synchrony, potentially as an extension of existing auto-AV delay algorithms.

**Visual Summary:**
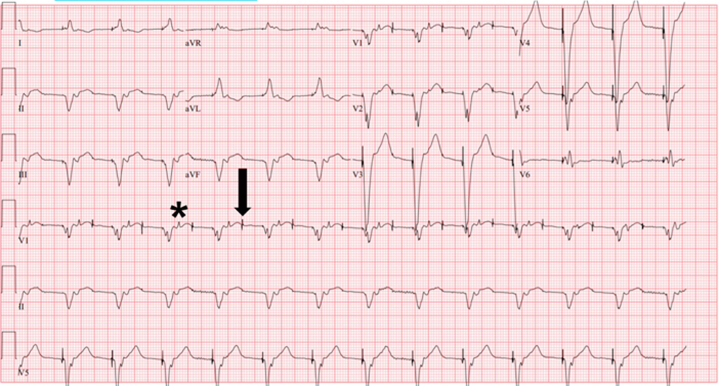
Presenting 12-Lead ECG With Ventricular Paced Rhythm at 80 beats/min Showing Retrograde Atrial Conduction (Asterisk) and Functional Atrial Noncapture (Arrow), Suggestive of RNRVAS Retrograde P waves are negative in leads II, III, and aVF; positive in leads I, aVL, and V_1_. ECG = electrocardiogram; RNRVAS = repetitive non-reentrant ventriculoatrial synchrony.

## Conclusions

RNRVAS is an underdiagnosed pacing-related arrhythmia that can cause significant hemodynamic compromise through functional AV dyssynchrony and promotion of atrial arrhythmias, often at lower heart rates that evade standard device detection algorithms. Careful analysis of device diagnostics and surface ECGs is essential to distinguish RNRVAS from PMT and pseudo-RNRVAS and to identify its contribution to heart failure exacerbations. Targeted device reprogramming, including adjustment of AV timing and LRLs, can effectively restore AV synchrony and provide meaningful symptomatic relief, as demonstrated in this case.

## Funding Support and Author Disclosures

This publication was supported by the National Center for Advancing Translational Sciences (NCATS; grant number UL1 TR002377). Its contents are solely the responsibility of the authors and do not necessarily represent the official views of the National Institutes of Health. The authors have reported that they have no relationships relevant to the contents of this paper to disclose.
